# Role of Aspartate 86 in the Catalytic Mechanism of *Escherichia coli* Glutamate Decarboxylase

**DOI:** 10.1021/acs.biochem.5c00666

**Published:** 2026-02-20

**Authors:** Fabio Giovannercole, Eugenia Pennacchietti, Gaia Grassini, Daniela De Biase

**Affiliations:** Department of Medico-Surgical Sciences and Biotechnologies, Sapienza University of Rome, Corso della Repubblica 79, 04100 Latina, Italy

## Abstract

In bacteria, the pyridoxal 5′-phosphate (PLP)-dependent enzyme glutamate decarboxylase (Gad) protects the cells exposed to an acidic environment by consuming one proton/catalytic cycle during the conversion of l-glutamate to γ-aminobutyrate (GABA) and CO_2_. The *Escherichia coli* enzyme (*Ec*GadB) is the best-characterized bacterial Gad; its activity is maximal at pH 4–5 and undetectable at pH ≥ 6.0, at which the active site is closed by His465. The imidazole ring of this His residue, highly conserved in bacterial Gad, becomes deprotonated as the pH increases above 5.0 and carries out a nucleophilic attack on the PLP-Lys276 Schiff base. However, when His465 is mutated, *Ec*GadB activity still displays pH dependence, indicating that other residues also play a role. Herein, through a combination of spectroscopic and kinetic analyses, including solvent kinetic isotope effect (SKIE) and proton inventory studies, Asp86, another residue highly conserved in bacterial Gad, was shown to play an important role in substrate binding and product release and, unexpectedly, to be a major player in the large SKIE observed in *Ec*GadB. This was demonstrated by incorporating the D86N substitution into the GadB_H465A variant to avoid the masking effect of His465 at pH > 5.5. In addition, GadB_D86N-H465A was shown to be less sensitive than GadB_H465A to the pH increase occurring during the decarboxylation, being still active in the pH range 7–8, where glutamate solubility increases. This finding, together with the enzyme’s improved ability to release the product, makes GadB_D86N-H465A interesting also for effective biobased synthesis of GABA.

## Graphical abstract

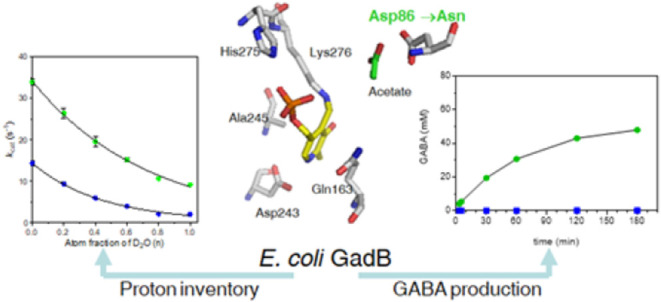

## Introduction

Glutamate decarboxylase (l-glutamate 1-carboxy-lyase, Gad; E.C. 4.1.1.15) is a ubiquitous pyridoxal 5′-phosphate (PLP)-dependent enzyme catalyzing the irreversible α-decarboxylation of l-glutamate (l-Glu) to yield γ-aminobutyrate (GABA) and CO_2_ (Scheme 1). In *Escherichia coli*, two biochemically indistinguishable isoforms, GadA and GadB, sharing 99% sequence identity and exhibiting an acidic pH optimum of activity (pH 3.8–5.0), are produced by the relevant genes.^1^ The GadB isoform (*Ec*GadB) is the most intensively studied at the biochemical and structural level;^2–6^ it is a 318 kDa homohexameric enzyme, whose pH-dependent activation can be easily monitored by a change in the absorption spectrum of its coenzyme, PLP.^5,6^ As shown in the dashed box in Scheme 1, in the absence of the substrate, the Lys276-PLP Schiff base typically undergoes interconversion between the active ketoenamine species (maximally absorbing at 420 nm) at acidic pH and the inactive substituted aldamine (maximally absorbing at 340 nm) at pH > 6.0. The substituted aldamine is the species in which the distal nitrogen of the imidazole ring of His465 forms a covalent bond with the PLP C4′ in the Lys276-PLP Schiff base. The interconversion between the active and inactive species is a highly cooperative pH-dependent process that occurs over a very narrow pH range (5.25–5.4), with a midpoint at pH 5.3. The midpoint of the spectroscopic transition shifts to pH 5.7 in the presence of chloride ions, which act as positive allosteric modulators of the enzyme.^3^

**Scheme 1:**
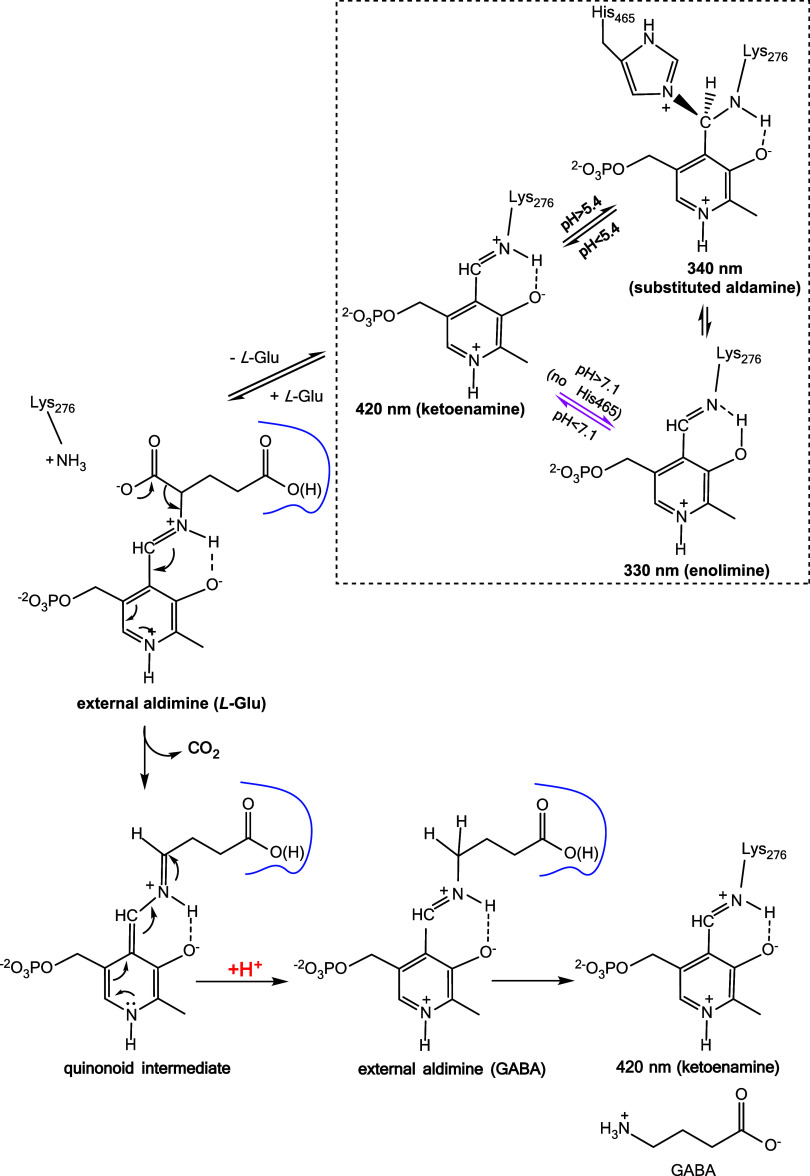
Intermediates in the Reaction Catalyzed by *Ec*GadB^a^

a The cofactor species at the active site of the enzyme in the absence of the substrate l-Glu are inside the dashed box. The magenta arrows indicate the equilibrium between the two cofactor species in the absence of His465. The blue curve indicates the enzyme active site pocket where Asp86 is located and where the binding of the γ-carboxylate of l-Glu occurs. The protonation state in the γ-carboxylate of l-Glu in the active site is not fully established: if deprotonated at acidic pH, other residues within the binding site must provide polar or positively charged groups to make this binding possible, while any group negatively charged will repel it, unless l-Glu is fully protonated.

The crystal structures obtained from the wild-type (wt) *Ec*GadB at different pH values and an N-terminal deletion mutant^2,3^ showed that three important local structural rearrangements occur in *Ec*GadB upon changes in pH: one affects the first 15 residues of the N-terminal domain (made up by the residues 1–57), the second affects residues 300–313, which form a β-hairpin in the PLP-binding domain (made up by the residues 58–346), and the third involves the last 15 residues (hereafter named C-tail) of the C-terminal domain (made up by the residues 347–466).

In the active form (pH < 5.5), in each subunit, the N-terminal residues fold into α-helices, the β-hairpin moves over the active site entry, and the C-tail becomes disordered (or highly flexible) and is not visible anymore in the crystal structure. In contrast, in the neutral-pH inactive form, the N-terminal residues unfold and contact the subunits, while the C-tail is ordered and, by slightly displacing the β-hairpin, “plugs” the active site and reversibly inactivates the enzyme via formation of the substituted aldamine (Scheme 1).^2^

All of the above findings fit nicely with the biological role assigned to bacterial Gad, which relieves the bacteria from the acid stress encountered in the environment, including during colonization of the gastrointestinal tract of the host.^7–9^ In fact, as shown in Scheme 1, Gad takes up one proton, which replaces the l-Glu α-carboxylate released as CO_2_. As a consequence of H^+^ consumption, the pH rises, and the enzyme activity naturally declines because the active ketoenamine species spontaneously converts into the inactive substituted aldamine species (Scheme 1, dashed box). Spectroscopic analysis of His465 variants demonstrated that this residue is key to the reversible inactivation of *Ec*GadB at acidic pH.^6^ On the contrary, His465 did not contribute to the cooperativity observed in the activity.^6^ However, the His465 variants, although still maximally active at pH 4.0–5.0, displayed a pH-activity profile broader than the wild-type enzyme, and their catalytic activity fell in the pH range 5.5–6.5, even though the ketoenamine form remained predominant.^6^ This led to the speculation that the decrease in the catalytic activity could be attributed to the deprotonation of one (or more) residue(s), with an expected p*K* of approximately 6.0. A likely candidate is the side chain carboxylate of Asp86, which, in the low-pH active form, was shown to be suitably oriented to bind the γ-carboxylate of l-Glu (Figure 1).^2^ This residue is provided by the neighboring subunit in the functional dimer (i.e., *Ec*GadB is in fact a trimer of dimers) and, in order to bind the γ-carboxyl group of l-Glu, it undergoes a significant side-chain reorientation during the pH-dependent conformational change that accompanies the activation of the enzyme at acidic pH. Only at acidic pH, the side chain of Asp86 was found to be oriented toward the active site (Figure 1).^2^

**Figure 1:**
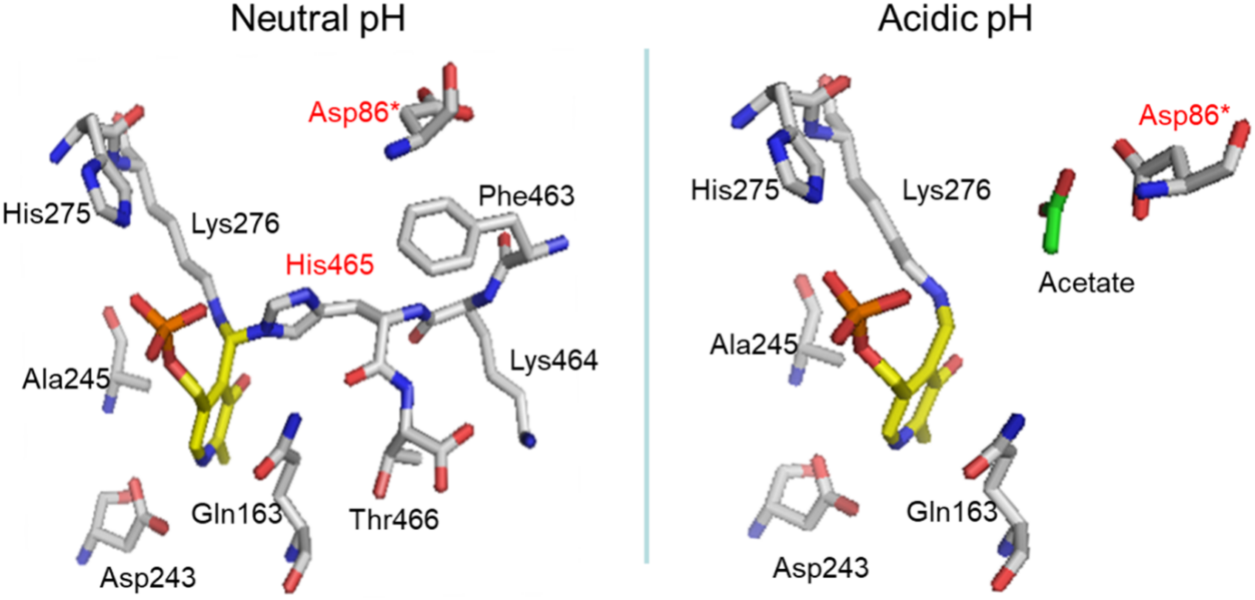
Details of the active site of EcGadB at neutral and acidic pH. Left: at pH 7.6 (PDB: 2DGK; closed conformation), the Asp86 side chain points away from the active site, and the C-terminal residues lock the active site, with His465 establishing an aldamine linkage with Lys276-PLP internal aldimine. Right: at pH 4.6 (PDB: 1PMM *;* open conformation), the Asp86 side chain moves into the active site and can interact with the distal carboxylate of l-Glu, whose position is mimicked by an acetate molecule found in the active site (acetate was present in the crystallization buffer). The asterisk refers to the residue that is contributed by the neighboring subunit in the functional dimer.

Clearly, for this interaction to occur, at least one of the two carboxylate groups must be protonated; otherwise, they would repel each other. Notably, an Asp residue was found to be strictly conserved in bacterial^7^ and plant Gad,^10^ both known to be active at acidic pH. Notably, in human Gad67/Gad65, the same role is structurally played by an arginine residue (Arg567/Arg558), and this is in accordance with the latter enzymes being active at neutral pH.^11^

In order to fully elucidate the role of this Asp residue, which is part of the bacterial Gad “signature”,^7^ a biochemical characterization of the GadB_D86N-H465A double variant was carried out in the present work. The double variant was required in order to fully assess the involvement of Asp86 in *Ec*GadB catalytic activity, which would be otherwise impossible when His465 is present because autoinactivation of *Ec*GadB occurs above pH 5.5 (Scheme 1, dashed box).^3,6^

In this work we provide evidence that Asp86 plays a role in product release and contributes to the large kinetic isotope effect observed in *Ec*GadB.^12,13^ Notably, the double variant catalyzes the decarboxylation of l-Glu at a significant rate in the pH range 7–8, a feature that we propose to be advantageous in applications where an effective biobased route of GABA synthesis is desirable.^6,14–17^

## Materials and Methods

### Materials

The GeneTailor Site-Directed Mutagenesis system (Invitrogen), d,l-Norvaline (Alfa Aesar), *Taq* DNA polymerase (used for colony screening of mutant clones), and sodium dodecyl sulfate-polyacrylamide gel electrophoresis (SDS-PAGE) protein markers were from Thermo Fisher Scientific. The DNA ligation system, prepacked HiPrep DEAE FF 16/10 (20 mL), and HiPrep 16/60 Sephacryl S-300 HR were from GE Healthcare. Kits for plasmid DNA purification and DNA extraction from agarose gel were from Macherey-Nagel. Ingredients for bacterial growth were from BD Difco (Fisher Scientific). Streptomycin sulfate was from U.S. Biochemical Corp. (Cleveland, OH, USA). Acetic acid and hydrochloric acid were from VWR International. *Taq Platinum* High-Fidelity DNA polymerase, restriction enzymes, alkaline phosphatase, GABase, ampicillin, kanamycin, l-glutamic acid, potassium dihydrogen phosphate, dipotassium hydrogen phosphate, sodium glutamate, sodium acetate, sodium chloride, deuterium oxide (D_2_O), GABA, glycerol, vitamin B6, and pyridoxal 5′-phosphate (PLP) were purchased from Merck. Vivaspin 500 (30 kDa) was from Sartorius Stedim Lab Ltd., UK. Oligonucleotide synthesis and DNA sequencing services were from Eurofins Genomics.

### Site-Directed Mutagenesis

Site-directed mutagenesis of *E. coli* GadB was carried out by overlap extension PCR on the entire plasmid pQ*gadB*.^1^ The single mutant plasmid pQ*gadB*-D86N was generated using two specific oligonucleotides: the forward (mutagenic) oligonucleotide 5′-CCATTAACAAAAACTGGATC*AAC* AAAGAAGAA-3′, which introduces the Asp → Asn mutation (GAC in wild-type *gadB*), and the reverse, partially complementary oligonucleotide 5′-GATCCAGTTTTTGTTAATGGACAAATCCAT-3′. The underlined and italicized sequences refer to the region where the two oligonucleotides overlap and the mutated codon, respectively. Plasmid pQ*gadB*_D86N-H465A was obtained by ligating a 771-bp *Nco*I-*Eco*RV DNA fragment (corresponding approximately to the first half of the gene) from plasmid pQ*gadB*_D86N into pQ*gadB*_H465A,^6^ previously digested with the same restriction enzymes to release the corresponding wild-type fragment. The newly generated plasmid was used to transform the *E. coli* strain JM109/pREP4. Transformed colonies were screened by colony PCR, and the positive clones were isolated. Plasmid pQ*gadB*_D86N-H465A was purified from the positive clones and sequenced on both strands to confirm the presence of only the two mutations.

### Expression and Purification of GadB_D86N-H465A

Expression and purification of GadB_D86N-H465A from *E. coli* JM109/pREP4/pQ*gadB*_D86N-H465A was essentially as described for wild-type GadB^1^ and GadB-H465A,^6^ except that the pH of the potassium phosphate buffer used during DEAE-Sepharose chromatography was pH 6.0 instead of 6.5, as GadB_D86N-H465A was found not to bind the resin at pH 6.5. Following the first DEAE chromatographic step, the enzyme was dialyzed against 0.1 M sodium acetate, pH 4.6, containing 0.1 mM dithiothreitol (DTT), concentrated to 25 mg/mL, and loaded (1 mL) onto a HiPrep 16/60 Sephacryl S-300 HR column equilibrated with the same buffer. Equilibration and chromatography were carried out at a flow rate of 0.5 mL/min. All of the chromatographic steps were carried out at 4 °C.

Wild-type GadB and GadB-H465A were purified for comparative analyses, following previously published protocols.^1,6^ Protein purity was analyzed by SDS-PAGE. The enzyme concentration was calculated as previously described.^1^ The PLP content of the purified enzymes was determined spectrophotometrically after treating each protein with 0.1 M NaOH. Under these conditions, PLP is released, and its molar absorption coefficient (ε) at 388 nm is 6.55 × 10^3^ M^–1^ cm^–1^.

### Gad-GABase Assay

*E. coli* Gad activity was assayed in 0.2 mL at 37 °C under different pH conditions, in either 50 mM sodium acetate buffer (pH 4.5–5.8) or 50 mM potassium phosphate buffer (pH 5.9–8.3) in the presence of 40 μM PLP, 50 mM NaCl, and 50 mM sodium glutamate. For each assay, 2–4 μg of enzyme was used. A 5–50 μL aliquot was withdrawn from the reaction mixtures at various time intervals, quenched at pH 8.6, and assayed with GABase, using a slightly modified protocol than previously described.^1^ Briefly, the reaction mixture was transferred to 100 μL of GABase solution containing 1.25 μL of activated GABase (0.02 U/μL), and the incubation was carried out for 60 min at 37 °C. The enzyme specific activity is given as μmol GABA min^–1^ mg^–1^ (U/mg).

GABA production at pH 7.0 was assayed over a period of 3 h. The reaction was conducted at 30 °C in 4 mL of 50 mM potassium phosphate buffer, pH 7.0, in the presence of 40 μM PLP and 50 mM sodium glutamate. Protein concentration was 2 μM (referred to as the monomer concentration). At specified time intervals, aliquots (100 μL) of the reaction mixture were withdrawn, quenched with 400 μL of 0.1 M *N*-(2-hydroxyethyl)­piperazine-*N*′-propanesulfonic acid (HEPPS), pH 8.6, and vigorously vortexed. A 5–50 μL aliquot was then analyzed for GABA content with the GABase assay, as above.

### Determination of Catalytic Constants and Solvent Kinetic Isotope Effects (SKIEs)

Catalytic constants and SKIEs on *k*_cat_ and *k*_cat_/*K*_M_ of the l-Glu decarboxylation catalyzed by wild-type *Ec*GadB, GadB_H465A, and GadB_D86N-H465A variants (0.075 μM in H_2_O or 0.151 μM in D_2_O) were determined in either 100% H_2_O or 97% D_2_O. The reaction was carried out at 25 °C in 250 μL of 50 mM sodium acetate buffer containing 40 μM PLP and different l-glutamate concentrations, ranging from 0.16 to 10 mM for wild-type *Ec*GadB and GadB_H465A or from 0.3 to 50 mM for GadB_D86N-H465A. SKIEs were measured in the pL (L = H or D) range 4.6–5.0 (Table 1). At various intervals, aliquots (50 μL) of the reaction mixture were withdrawn and quenched in 200 μL of 0.1 M HEPES–NaOH, pH 8.6, and the GABA content was analyzed by the GABase assay (see above). For some confirmatory experiments, GABA was quantified by high-performance liquid chromatography (HPLC), as described elsewhere.^18,19^

**Table 1:** Midpoint (p*K*) and Hill Coefficient (*n*) of the Spectroscopic Transition in Wild-Type (wt) and Variant Forms of EcGadB

	p*K*	*n*	isosbestic point (nm)
GadB wt	5.31 ± 0.01	10.52 ± 1.49	360
GadB wt + NaCl	5.70 ± 0.01	10.14 ± 1.04	360
GadB_H465A	7.44 ± 0.01	0.60 ± 0.05	348
GadB_D86N-H465A	7.23 ± 0.14	0.77 ± 0.17	360

For the experiments performed in buffers prepared with D_2_O, the pH values of the buffer solutions were adjusted according to the following relationship: pD = pH + 0.4.

### Proton Inventory

A proton inventory on *k*_cat_ for wild-type *Ec*GadB- and GadB_D86N-H465A-catalyzed reactions was performed by varying the atom fraction of D_2_O at pL 4.8. The reaction conditions were the same as those for the SKIEs, except that for the proton inventory, on *k*_cat_, sodium glutamate was 50 mM for *Ec*GadB and 80 mM for GadB_D86N-H465A. GABA was quantified using the GABase assay (see above).

The best fitting for the proton inventories on *k*_cat_ was obtained using eq 1 for GadB_D86N-H465A and using eq 2 for wild-type GadB.

1 $$k_{n}=k_{0}{\varphi_{i}}^{\mathrm{TS}}$$

$$k_{n}=k_{0}\left[f\prod_{i=1}^{m_{i}}\left(1-n+n{\varphi_{i}}^{\mathrm{TS}}\right)+\left(1-f\right)\prod_{j=1}^{m_{j}}\left(1-n+n{\varphi_{j}}^{\mathrm{TS}}\right)\right]$$ 2

In both equations, *k_n_* is *k*_cat_ at each atom fraction of D_2_O (*n*), while *k*_0_ is *k*_cat_ in 100% H_2_O. φ^TS^ represents the transition-state isotopic fractionation factor, whereas *f* and (1 – *f*) are the fraction contributions to φ*_i_* ^TS^ and φ*_j_* ^TS^, respectively. Both equations are simplified versions of the Gross–Butler equation,^20^ where a single pathway contributes with an “infinite” proton transfer (eq 1) or where two concomitant pathways contribute in parallel to proton transfer (eq 2). Moreover, the reactant-state fractionation factor (φ^RS^) in the Gross–Butler equation was assumed to be 1, as usually reported for proteins^20^ and in agreement with previous findings.^13^

### Spectrophotometric Measurements and Data Analysis

Absorption spectra were recorded at the indicated temperatures using an Agilent model 8453 diode array spectrophotometer. The absorption spectra were resolved into their component absorption bands (deconvolution) by a nonlinear least-squares fit of the experimental data to the sum of a variable number of log-normal curves, each having independent parameters.^21^ Deconvolution of spectra was performed with Scientist (Micromath, Salt Lake City, UT).^22^

Curve fitting and statistical analyses were performed using GraphPad Prism 8.2.10 (GraphPad Software, San Diego, CA). The pH-dependent variation in absorbance and activity of the wild-type and enzyme variants was analyzed using the Hill equation (eq 3)­

3 $$Y=Y_{\mathrm{Bottom}}+\frac{\left(Y_{\mathrm{Top}}-Y_{\mathrm{Bottom}}\right)}{1+10^{\left(pK-\mathrm{pH}\right)n}}$$

where *Y*_Bottom_ is the *Y* value at the bottom plateau, *Y*_Top_ is the *Y* value at the top plateau, p*K* is the pH value at the midpoint of the sigmoid, and *n* is the Hill coefficient corresponding to the (minimum) number of protons involved in the transition.

## Results

### Purification and Spectroscopic Properties of GadB_D86N-H465A

The GadB_D86N-H465A variant was generated by incorporating the D86N mutation into the single variant GadB_H465A already available in the laboratory.^6^ GadB_D86N-H465A was overexpressed in *E. coli* and purified as previously described^6^ except for ion-exchange chromatography, which was carried out in phosphate buffer at pH 6.0, since GadB_D86N-H465A was found not to bind the resin at pH 6.5. During chromatography, a yellow band was observed on the column, indicating that, like GadB_H465A, at pH 6.0–6.5, the PLP-Lys276 Schiff base in GadB_D86N-H465A is mostly in the 420 nm-absorbing form, i.e., the ketoenamine (Scheme 1, dashed box, and Figure 1, left panel). Moreover, the fractions containing purified GadB_D86N-H465A had a 70% PLP complement. Therefore, to attain a full complement of PLP, an incubation for 1 h at room temperature in the dark with a fivefold molar excess of PLP relative to the protein concentration was carried out. Following dialysis in acetate buffer at pH 4.6, the double variant was further purified by gel filtration chromatography. The protein was judged ≥95% pure by SDS-PAGE (data not shown), and the PLP content was 90–95% (i.e., the PLP content varied slightly from purification to purification but was never below 90%), a value slightly lower than that found in wild-type *Ec*GadB (Figure 2). The yield of the purified enzyme from a standard purification (2 L of bacterial culture) was 30 mg. The specific activity toward l-Glu (assayed at 37 °C in 0.2 M pyridine/HCl buffer, pH 4.6, containing 1 mM PLP and 0.1 mM DTT) was 140 U/mg, corresponding to 64% and 80% of the specific activity of wild-type *Ec*GadB and GadB_H465A, respectively.^6^

**Figure 2:**
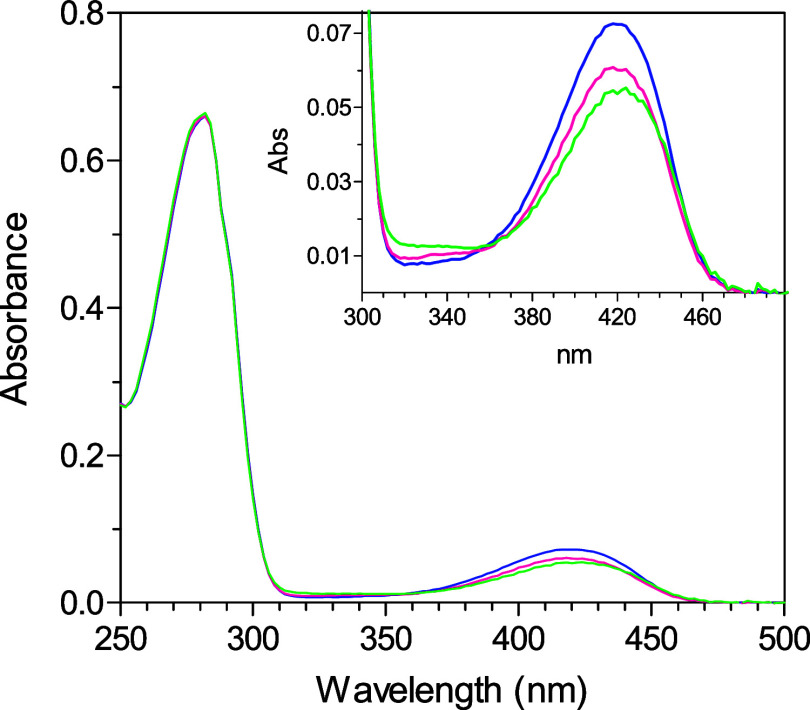
UV–visible absorbance spectra. The spectra of GadB_D86N-H465A (green), wild-type *Ec*GadB (blue), and GadB_H465A (magenta) were recorded in 50 mM sodium acetate buffer, pH 4.6. The protein concentration was 7.7 μM (referred to as the monomer; 0.41 mg/mL). Inset: expanded view of the 300–500 nm region of coenzyme absorbance.

When assayed with aspartate or glutamine, GadB_D86N-H465A did not show any activity, indicating that the D86N mutation in the active site of *Ec*GadB did not affect the substrate specificity of the enzyme with respect to these amino acids.

The UV–visible absorption spectra of the GadB_D86N-H465A variant, recorded at pH 4.5–9.1, provided the first indication of a pH-dependent 420 → 332 nm spectroscopic transition. Although the species absorbing at 332 nm prevailed at pH 9.1, the ketoenamine species was still detectable at this pH (Figure 3A).

**Figure 3:**
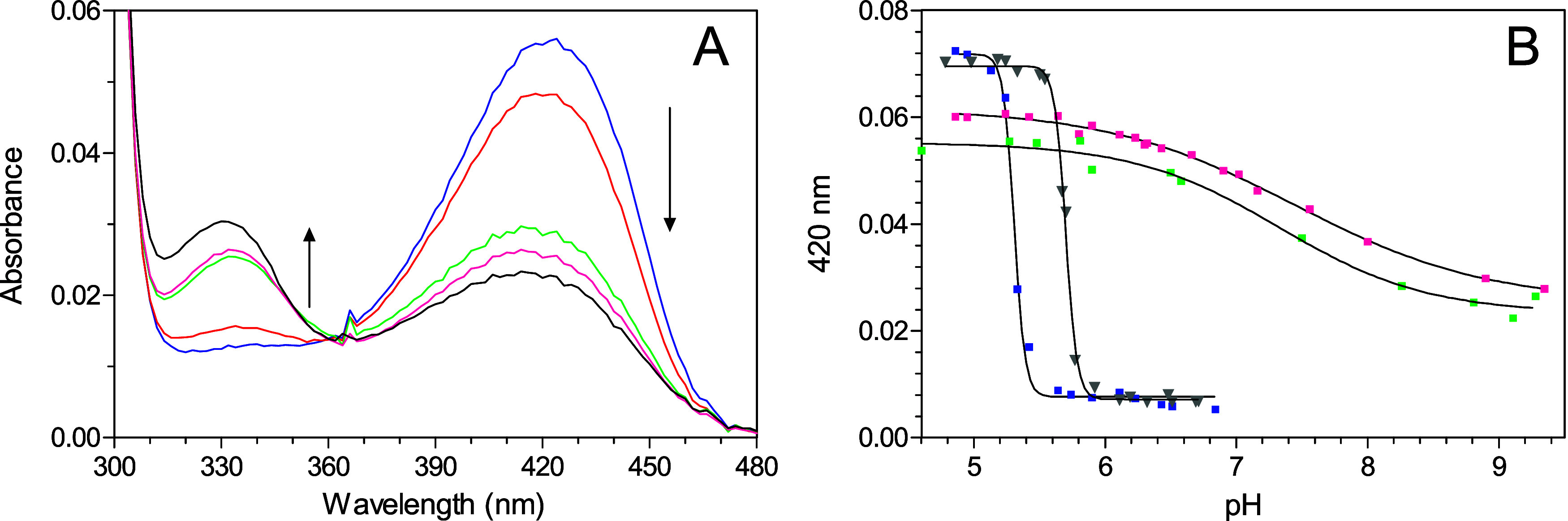
pH-dependent absorbance changes in GadB-D86N-H465A. (A) Absorption spectra of GadB-D86N-H465A were recorded in 50 mM sodium acetate buffer from pH 4.6 (blue) to pH 5.48 (not shown because it is identical to the spectrum at pH 4.6) and in 50 mM potassium phosphate buffer at pH 6.58 (red), 8.26 (green), 8.81 (magenta), and 9.1 (black). The protein concentration was 7.7 μM. The arrows indicate the direction of the change in absorbance at 420 and 332 nm upon increasing pH. (B) pH-dependent readings at 420 nm, in the above buffer systems, are reported for GadB_D86N-H465A (green squares), GadB_H465A (magenta squares), and wild-type *Ec*GadB in the absence (blue squares) and presence (gray inverted triangles) of 50 mM NaCl. The solid lines through the experimental points represent the theoretical curves obtained fitting the experimental points with the Hill equation (eq 3 in the Materials and Methods).

This spectroscopic transition was significantly different from that occurring in wild-type *Ec*GadB,^5,6^ while it resembled that occurring in GadB_H465A, thus suggesting that, alike the GadB_H465A variant, the GadB_D86N-H465A variant retains activity at near neutral pH (see more details in section Catalytic Properties: Effect of pH on Specific Activity).^6^ As a matter of fact, when the 420 nm absorbance readings at each pH value were fitted with the Hill equation, the titration curves (Figure 3B) showed that the midpoint of the spectroscopic transition (p*K*) and the degree of the cooperativity, provided by the Hill coefficient (*n*), were similar across the two variants (Table 1).^6^ Notably, the p*K* was two pH units higher than that observed for wild-type *Ec*GadB and the degree of cooperativity was abolished.

As previously suggested for the GadB_H465A variant,^6^ a negative cooperativity can be explained by fitting the experimental data to an equation in which the p*K* value of an ionizable group involved in the equilibrium between the 420 and 332 nm-absorbing forms increases as a result of the conformational shift. This kind of p*K* alteration in an ionizable group commonly occurs in catalytic residues within enzyme active sites and is referred to as the Born (or desolvation) effect, which promotes the neutral state of a titratable group as it moves from a polar to a nonpolar environment.^23^ Examination of the His465 variants showed that a residue in the active site experiences this p*K* shift when the surroundings of GadB’s active site become less polar.^6^ The current data on the GadB_D86N-H465A variant suggest that, because the same is observed in this variant, the H465A mutation is mainly responsible for the observed findings.

Moreover, chloride ions, known to affect the spectroscopic transition and to act as positive allosteric activators of *Ec*GadB (Figure 3B and Table 1),^3^ did not exert any effect on GadB_D86N-H465A (data not shown). The results obtained with the GadB_D86N-H465A variant resembled those obtained with GadB_H465A (Figure 3B and Table 1), except for the isosbestic point of the spectroscopic transition, which in GadB_D86N-H465A occurred at 360 nm (Figure 3A and Table 1), as in wild-type *Ec*GadB, whereas it was blue-shifted to 348 nm in GadB_H465A (Table 1 and ref 6).

In order to identify more precisely the species contributing to the absorbance spectra, the spectrum at pH 8.8 of GadB_D86N-H465A was resolved into single-component absorption bands (Figure 4) by a nonlinear least-squares fit (deconvolution).

**Figure 4:**
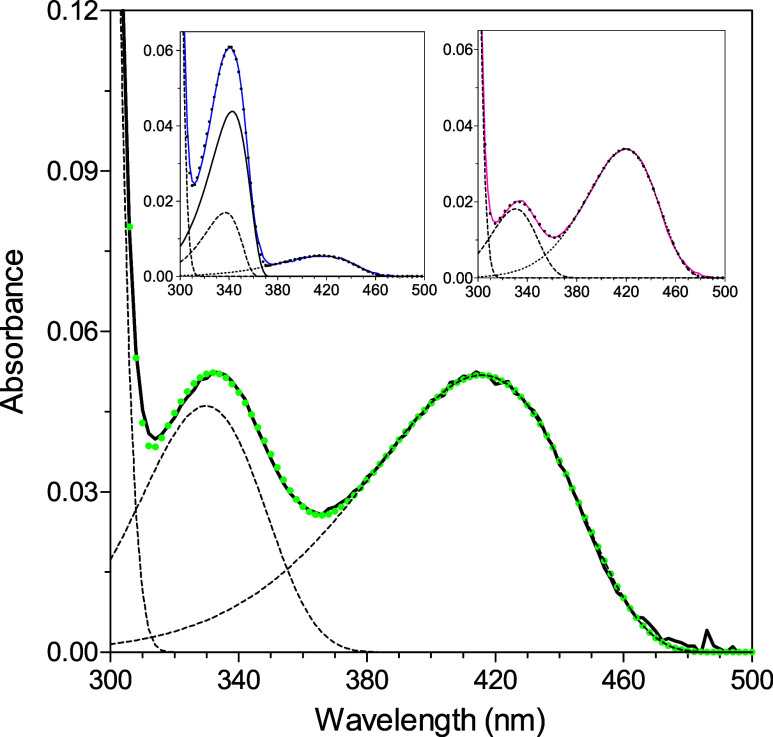
Deconvolution of absorption spectra. The absorbance spectrum of GadB_D86N-H465A at pH 8.8 was resolved into its component absorption bands. For comparison, in the insets are reported the spectra of wild-type *Ec*GadB at pH 6.84 (inset on the left) and GadB_H465A at pH 8.8 (inset on the right) resolved into their component absorption bands. The spectra were recorded in a 50 mM potassium phosphate buffer. The solid lines represent the experimental spectra, the dashed lines are the component bands, and the dotted colored curve is the theoretical curve obtained by a nonlinear least-squares fit of the experimental points.^22^

It is important here to point out that in wild-type *Ec*GadB, the spectrum at neutral pH (or higher), where the enzyme is inactive, arises from the sum of three species, with absorption maxima at 330, 341.5, and 410 nm (Figure 4, inset on the top left); in particular, the 341.5 nm component band, corresponding to the substituted aldamine (see Scheme 1 and ref 6) prevails over the other two species at neutral pH. On the contrary, spectral deconvolution of GadB_D86N-H465A yielded only two species with absorption maxima at 330 nm (enolimine) and 418 nm (ketoenamine) (Figure 4 and Scheme 1), regardless of whether the pH was 4.6 (data not shown) or 8.8 (Figure 4). This analysis confirmed that aldamine is absent in GadB_D86N-H465A, as observed in GadB_H465A.^6^ The only difference observed between the double and the single variant resided in the intensity of the absorbance at pH 8.8 of the 330 nm-absorbing enolimine species, which was higher in GadB_D86N-H465A than in GadB_H465A (Figure 4 vs the inset on the top right).

In conclusion, the spectroscopic properties of GadB_H465A and GadB_D86N-H465A turned out to be similar, indicating that the H465A mutation is the one responsible for the replacement of the substituted aldamine in the *Ec*GadB active site by the enolimine tautomer in the two variants (Scheme 1, dashed box).

### Catalytic Properties: Kinetic Constants and Solvent Kinetic Isotope Effect

Solvent kinetic isotope effects (SKIEs) on *k*_cat_ and *k*_cat_/*K*_M_ of l-Glu decarboxylation catalyzed by wild-type *Ec*GadB, GadB_H465A, and GadB_D86N-H465A variants were determined using buffers prepared in either 100% H_2_O or 97% D_2_O. The reactions were conducted at 25 °C over a range of l-Glu concentrations (see Materials and Methods). SKIEs were measured across a pL (L = H or D) range of 4.6–5.0 (Table 2). This range was selected because *Ec*GadB and GadB_H465A exhibit maximal activity within this interval.^6^ At pH 4.4–5.0, the GadB_D86N-H465A variant remained stable and maximally active under our assay conditions.

**Table 2:** Kinetic Parameters of the l-Glu Decarboxylation Catalyzed by Wild-Type *Ec*GadB, GadB_H465A, and Gad_D86N-H465A at Different pL

		H_2_O			D_2_O		
*Ec*GadB	pL	*k*_cat_ (s^–1^)	*K*_M_ (mM)	*k*_cat_/*K*_M_ (s^–1^ mM^–1^)	*k*_cat_ (s^–1^)	*K*_M_ (mM)	*k*_cat_/*K*_M_ (s^–1^ mM^–1^)
wild-type	4.60	14.21 ± 0.39	1.040 ± 0.110	13.66 ± 1.50	2.05 ± 0.06	0.258 ± 0.031	7.93 ± 0.98
	4.80	17.03 ± 0.48	1.234 ± 0.129	13.80 ± 1.50	1.99 ± 0.11	0.241 ± 0.057	8.26 ± 2.00
	5.00	14.25 ± 0.42	0.866 ± 0.089	16.46 ± 1.76	1.86 ± 0.06	0.265 ± 0.037	6.97 ± 1.01
H465A	4.60	17.02 ± 0.52	0.819 ± 0.103	20.78 ± 2.60	2.51 ± 0.15	0.281 ± 0.062	8.93 ± 2.41
	4.80	15.57 ± 0.36 (5.34 ± 0.24)^a^	0.767 ± 0.066 (6.18 ± 1.02)^a^	20.30 ± 1.77 (1.16 ± 0.15)^a^	2.33 ± 0.09	0.240 ± 0.037	9.71 ± 1.53
	5.00	17.83 ± 0.88	0.982 ± 0.166	18.16 ± 4.00	2.58 ± 0.09	0.175 ± 0.026	14.74 ± 2.26
D86N-H465A	4.60	29.06 ± 1.13	3.655 ± 0.596	7.95 ± 1.33	10.96 ± 0.37	1.435 ± 0.182	7.64 ± 1.00
	4.80	29.81 ± 0.74 (5.86 ± 0.15)^a^	4.143 ± 0.382 (5.56 ± 0.53)^a^	7.20 ± 0.69 (1.05 ± 0.10)^a^	10.23 ± 0.36	3.011 ± 0.373	3.40 ± 0.41
	5.00	27.66 ± 0.60	3.325 ± 0.333	8.32 ± 0.85	7.39 ± 0.21	1.081 ± 0.135	6.84 ± 0.88

a The kinetic parameters in parentheses were obtained at pL 6.2.

To mimic the viscosity effect caused by D_2_O, viscosity control was performed by assaying the enzyme activity in a buffer containing 9% (w/w) glycerol. These assays provided evidence that viscosity has no effect on the reaction rate (data not shown).

As shown in Table 2, the *K*_M_ and *k*_cat_ of the decarboxylation reaction catalyzed by GadB_D86N-H465A were both higher than those of wild-type *Ec*GadB and GadB_H465A, but the specificity constant (*k*_cat_/*K*_M_) turned out to be lower than those of wild-type *Ec*GadB and GadB_H465A because of the more marked increase of *K*_M_. The increase of *K*_M_ (Table 2) in the double variant is likely due to the substitution of Asp86, a key residue in the active site, highly conserved in bacterial Gads, which is provided by the neighboring subunit in the functional dimer and is significantly changing its orientation during the transition from the acidic to the neutral pH form (see Figure 1).

Unlike wild-type *Ec*GadB, the single variant GadB_H465A could be compared to the double variant GadB_D86N-H465A because the active sites of both were still accessible at pH > 6.0 (Figure 3),^3,6^ i.e., when the deprotonation of the side chain carboxylates of Asp86 and l-Glu would not favor their interaction. Thus, by comparing the *K*_M_ and *k*_cat_ of the decarboxylation reaction catalyzed by both enzyme variants at pH > 6.0, the role of Asp86 as a residue primarily responsible for either substrate binding or catalysis could be assessed. As indicated by the values in parentheses in Table 2, at pH 6.2, both variants attained similar *K*_M_’s. However, in the single variant, the increase was more pronounced (eight times) than in the double variant (1.8 times). Based on these results, it can be concluded that the decrease in the activity at pH 6.2 (see also section Catalytic Properties: Effect of pH on Specific Activity) is more likely due to a change in the orientation of the residue at position 86 (as a consequence of a conformational change in the active site) rather than to the nature of the side chain itself (i.e., neutral for Asn and negatively charged for Asp).

The decarboxylation reactions in wild-type *Ec*GadB, GadB_H465A, and GadB_D86N-H465A were also carried out in deuterium oxide (D_2_O) (Table 2, last three columns), and the *k*_cat_ and *K*_M_ values were calculated. In analogy with previous studies that used isotopically labeled l-Glu,^13^ the *k*_cat_ values in H_2_O and D_2_O for wild-type *Ec*GadB were found to be significantly different, giving rise to a solvent kinetic isotope effect (^D_2_^ ^O^ *k*_cat_) of 7–8, a rarely observed phenomenon in enzymes, including PLP-dependent ones.^24–26^ This effect was proposed to arise from the additive contributions of solvent-exchangeable protons at the transaldimination step (i.e., Schiff-base interchange, Scheme 1) and, later, at the decarboxylation step, with a conformational change possibly involved.^13^ The data in Table 2 indicate that the ^D_2_^ ^O^ *k*_cat_ of the decarboxylation reaction catalyzed by *Ec*GadB remained essentially unchanged in the single variant GadB_H465A (^D_2_O^ *k*_cat_ approximately 7), whereas it was reduced by more than half in the double variant GadB_D86N-H465A, decreasing to approximately 3.

Because the *k*_cat_ value contains rate constants for all steps following substrate binding, including the release of GABA (see Scheme 2 in Discussion for details), the large decrease in ^D_2_O^ *k*_cat_ in the *Ec*GadB- and in GadB_H465A-catalyzed reactions indicates that Asp86, still present in both enzymes, plays a key role also in the catalytic steps following substrate binding. This is an unexpected finding since Asp86 was initially thought to affect catalysis by contributing to substrate binding, depending on the pH-dependent protonation state of its side chain carboxylate.

**Scheme 2:**
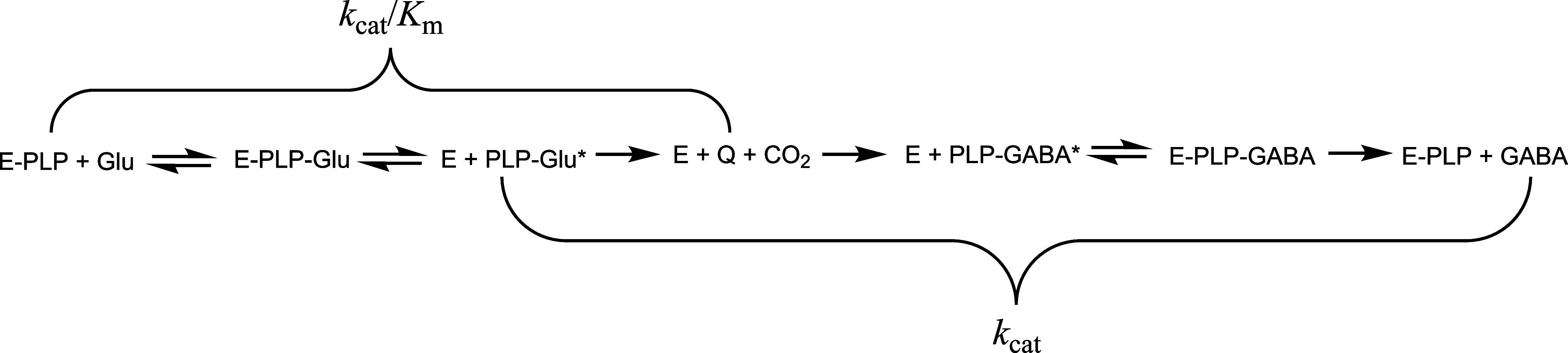
Catalytic Steps in the Decarboxylation of l-Glu in *Ec*GadB^a^

a Q is the quinonoid intermediate, i.e., the short-living cofactor–substrate intermediate after decarboxylation, which is then quickly reprotonated, thus generating the external aldimine with GABA (PLP-GABA*). Upon transaldimination, E-PLP-GABA complex generates E-PLP and GABA, which then leads to the release of the product (GABA). Notably, GABA is neither a substrate nor an inhibitor of *Ec*GadB.^12^

### Proton Inventory

A proton inventory study was conducted to obtain additional information on the solvent isotope effect on *k*_cat_ for the l-Glu decarboxylation reaction catalyzed by wild-type *Ec*GadB and GadB_D86N-H465A. The GadB_H465A variant was not included in this study because the kinetic constants of its reaction in both H_2_O and D_2_O mirror those of wild-type *Ec*GadB (Table 2). Experiments were performed at a pL of 4.8 since the *k*_cat_ versus pL profiles for wild-type and GadB_D86N-H465A are relatively independent of pL over the range 4.6–5.0, as shown in Table 2. The ^D_2_O^ *k*_cat_ was studied at 50 and 80 mM l-Glu for wild-type *Ec*GadB and GadB_D86N-H465A, respectively. These concentrations are well above the *K*_M_ values calculated in both H_2_O and D_2_O buffers for each enzyme (Table 2). For wild-type *Ec*GadB, the proton inventory revealed a pronounced downward-bowing curve (hypercurvature), which indicates the occurrence of multiple proton transfers contributing to ^D_2_O^ *k*_cat_ (Figure 5), in line with previous reports.^13^

**Figure 5:**
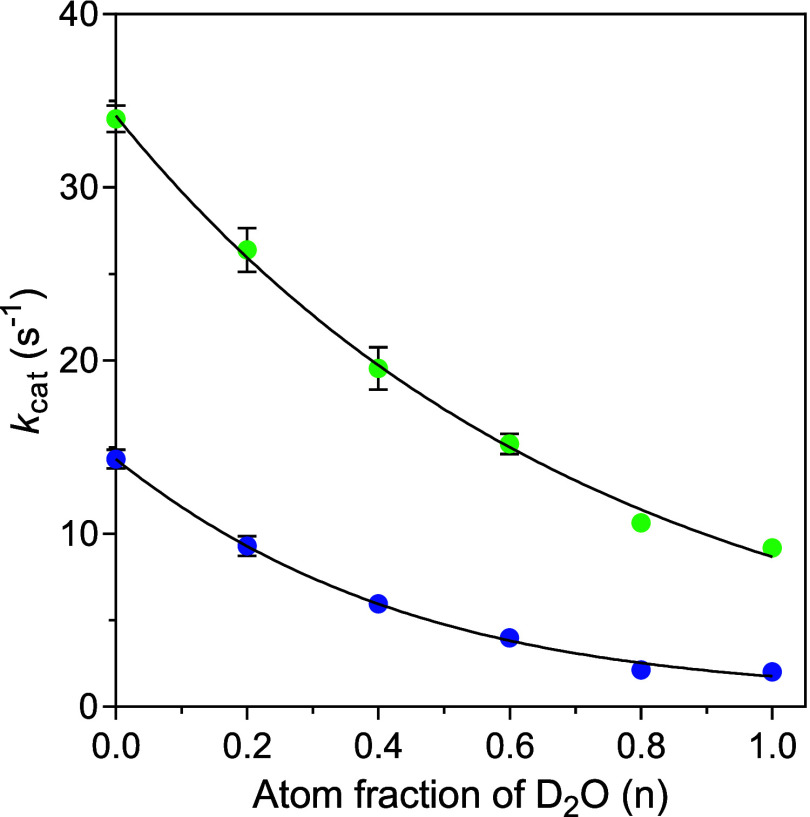
Proton inventory for wild-type *Ec*GadB (blue) and GadB_D86N-H465A (green), showing solvent deuterium dependence of *k*_cat_ on the fraction of deuterium (*n*) in the solvent. Reactions were carried out at a pL of 4.8 in the presence of a saturating concentration of l-Glu, i.e., 50 mM for wild-type *Ec*GadB (blue) and 80 mM for GadB_D86N-H465A (green). Data were fitted to eq 1 for GadB**_**D86N-H465A and eq 2 for wild-type *Ec*GadB.

Unlike what was previously suggested,^13^ the best nonlinear fit was obtained using eq 2 (Materials and Methods) with a “multichannel” model assuming the reactant-state fractionation factor (φ^RS^) in the Gross–Butler equation equals 1 and predicting contributions from two transition-state fractionation factors: φ*_i_* ^TS^ = 0.1003 ± 0.0327, which contributes with an “infinite” number of protons to the overall isotopic effect; φ*_j_* ^TS^ = 0.5986, which was fixed based on the obtained values of SKIE for *Ec*GadB (Table 2), contributing with four protons to the overall isotopic effect. The first pathway is expected to contribute 96 ± 9%, while the second pathway contributes 4 ± 9%, giving rise to an isotope effect of 8.30 ± 0.60, slightly lower but consistent with the SKIE value of 8.56 ± 0.95, similar to the ^D_2_O^ *k*_cat_ reported above (Table 2).

The proton inventory for GadB_D86N_H465A also revealed a downward-bowing curve, but much less pronounced than the wild-type *Ec*GadB curve (Figure 5). The former curve was easily fit by several forms of eq 1 (Materials and Methods), revealing the involvement of only one transition-state fractionation factor. The best fit was obtained with a model predicting an “infinite” number of transition-state protons (φ^TS^ = 0.2537 ± 0.0098) that accounts for an isotopic effect of 3.94 ± 0.15, slightly higher but consistent with the SKIE value (2.91 ± 0.07). Transition-state hydrogen bridges in solvation catalysis are likely responsible for this isotopic effect.^20^ Hence, the effect of the D86N substitution consists of suppressing the multichannel model observed in wild-type *Ec*GadB, which leads to an overall decrease of the isotope effect by 2.1-fold.

### Catalytic Properties: Effect of pH on Specific Activity

All of the above findings suggest that Asp86 in *Ec*GadB plays a negative role in GABA release but positively affects the substrate binding at acidic pH, as originally supposed.^2^ It was thus hypothesized that the replacement of Asp86 with an Asn residue, as in GadB_D86N-H465A, could be exploited for GABA synthesis at pH > 7.0, where wild-type *Ec*GadB is totally inactive and the single variant GadB_H465A is drastically affected.^6^ Thus, the activity profile of GadB_D86N-H465A in the pH range 4.5–8.3 was compared with those of wild-type *Ec*GadB and GadB_H465A (Figure 6A). The results showed that GadB_D86N-H465A retained activity at pH > 7, where wild-type *Ec*GadB and GadB_H465A activity were undetectable (Figure 6A). Notably, GadB_D86N-H465A retained 10 and 6% of the starting activity at pH 7.0 and 8.0, respectively. The cooperativity in the activity of GadB_D86N-H465A is almost abolished and resembles that of GadB_H465A.^6^ The changes in the cooperativity detected by analyzing the spectroscopic transitions (ref 6 and Figure 3B) and by measuring the activity clearly reflect the different experimental approaches, i.e., absence in the former and presence in the latter of the l-Glu. Given that GadB_D86N-H465A was still active at pH 7.0–8.0, GABA production was measured in phosphate buffer at pH 7.0 in the presence of 50 mM l-Glu over a period of 3 h (Figure 6B). For comparative purposes, the time course of GABA production was also analyzed for GadB_H465A and wild-type *Ec*GadB. As shown in Figure 6B, GadB_D86N-H465A converted 86% of l-Glu into GABA after 2 h and 96% after 3 h of reaction, leaving 7 and 2 mM residual l-Glu at each time point, respectively.

**Figure 6:**
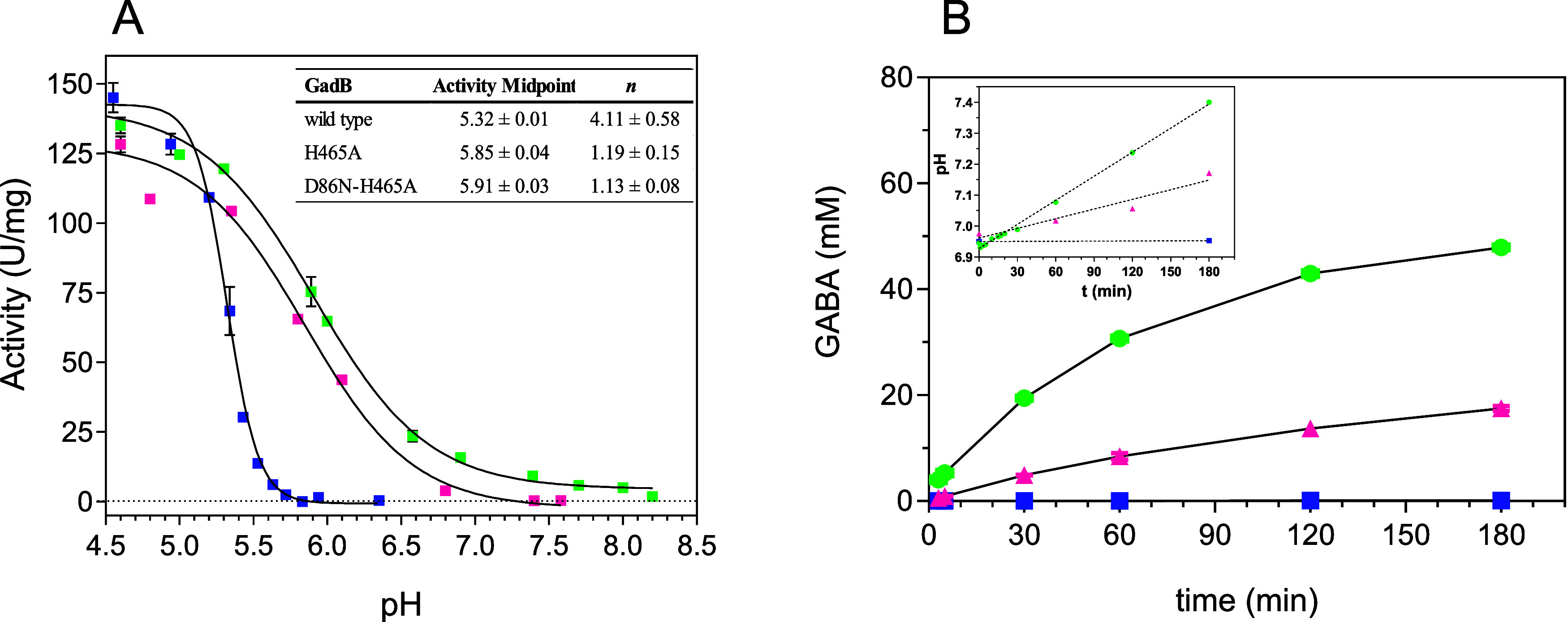
Effect of pH on the specific activity and time course of GABA production. Blue symbols, wild-type *Ec*GadB; magenta symbols, GadB_H465A; and green symbols, GadB-D86N-H465A. (A) Activity assays were carried out at 37 °C in 50 mM acetate (pH 4.5–5.8) or phosphate (pH 5.9–8.3) buffer in the presence of 40 μM PLP, 50 mM NaCl, and 50 mM l-Glu. The protein concentration was 0.9–2 μM. The solid lines through the experimental points represent the theoretical curves obtained using the Hill equation (Materials and Methods). The reported data are the means of three independent experiments, with a standard deviation <10% of the given value. The *n* and activity midpoint values retrieved from curve fitting with the Hill equation are given in the inset table. (B) GABA production was analyzed over a period of 3 h. The reaction was carried out at 30 °C in 4 mL of 50 mM potassium phosphate buffer, pH 7.0, containing 40 μM PLP and 50 mM l-Glu. The protein concentration was 2 μM. At each time point, aliquots (100 μL) were withdrawn and analyzed for GABA content using the GABase assay. The pH changes during the reaction were also recorded (inset). The reported data are the means of two independent experiments, with a standard deviation <5% of the given value.

After the first 30 min of reaction, the rate of GABA production gradually decreased due to a pH increase, although it did not stop in either variant. However, the GadB_D86N-H465A variant showed a significantly reduced sensitivity to pH increase (Figure 6B inset), which allowed us to obtain an almost complete conversion of l-Glu into GABA.

These results are remarkable when compared with those obtained with GadB_H465A, which converted only 35% of l-Glu into GABA after 3 h (Figure 6B). As expected, wild-type *Ec*GadB decarboxylated only <1% of l-Glu after 3 h at pH 7.0. During the reaction, the pH of the solutions was monitored: after 3 h, the pH increased by 0.2 and 0.4 pH units in GadB_H465A and GadB_D86N-H465A reactions, respectively (Figure 6B, inset). This is consistent with H^+^ consumption during the decarboxylation reaction in a buffer system at 50 mM, i.e., similar to that of the substrate. In wild-type *Ec*GadB, no pH change was observed because the enzyme was inactive. In conclusion, the D86N mutation leads to a noticeable expansion of *Ec*GadB activity toward alkaline pH when incorporated in the GadB_H465A variant.

## Discussion

In many enteric bacteria, the enzyme Gad is a major structural component of a very potent acid-resistance system, i.e., the glutamate-dependent acid-resistance system, which protects these microorganisms from extremely acidic stress, such as that encountered during transit through the host stomach.^7^ Examples of bacteria employing this system include commensal and pathogenic strains of *E. coli*, *Shigella flexneri*, *Listeria monocytogenes*, *Brucella microti* and several other Brucella species (among those evolutionary most ancient), *Lactobacillus reuteri*, and *Lactococcus lactis*, including human intestinal *Bacteroides* spp.^27–29^ Therefore, it is not surprising that many residues are strictly conserved in bacterial Gad, suggesting that this enzyme in bacteria has evolved to accomplish the proton-consuming decarboxylation reaction only when the intracellular pH falls below a life-threatening threshold level, as a consequence of the low external pH and proton “leakage” into the cells.^28^ Clearly, some amino acid residues in the Gad “signature” ^7,9^ are of crucial importance from the functional point of view. Asp86 was hypothesized to be among those. In this work, the role of Asp86 in *Ec*GadB activity was therefore investigated in detail by examining the spectroscopic and catalytic properties of the enzyme. To do so, Asp86 was substituted with Asn, a residue of similar size bearing a polar but neutral side chain. This mutation was expected not to affect the hydrophilicity in the *Ec*GadB active site and steric hindrance while eliminating the charge repulsion between the β-carboxylate of Asp86 and the γ-carboxylate of l-Glu under nonpermissive pH conditions (i.e., at pH > 5.5). As a consequence of the mutation D86N, substrate binding was expected to be still possible at pH values above 6.

To assess the role of Asp86 in *Ec*GadB, we produced a double variantof GadB_D86N-H465A. This was necessary to evaluate the contribution of Asp86 in substrate binding and the catalytic mechanism at pH > 5.5, an interaction otherwise undetectable due to the presence of His465 in the active site at pH > 5.5. While the D86N mutation in the double variant GadB_D86N-H465A does not significantly affect the spectroscopic properties with respect to the single variant GadB_H465A (Figures 3B and 4 and Table 1), the data obtained in this work provide evidence that the l-Glu decarboxylation reaction catalyzed by GadB_D86N-H465A (i) displays a significantly reduced SKIE and (ii) has an expanded pH range of activity with respect to both wild-type *Ec*GadB and GadB_H465A.

In order to discuss the large differences in the SKIE for the wild-type *Ec*GadB and GadB_D86N-H465A double variant, the model in Scheme 2 for the reaction catalyzed by *Ec*GadB can be considered. This model aligns with those published previously by others^13,30,31^ and reflects the catalytic steps of the Gad reaction.

In the model, l-Glu binds to the internal aldimine (E-PLP) to generate, via transaldimination (E-PLP-Glu), the external aldimine that positions the α-carboxylate of l-Glu in a catalytically competent orientation, perpendicular to the planar π system of the external aldimine (PLP-Glu*). This permits decarboxylation followed by reprotonation of the quinonoid intermediate (Q) to generate the external aldimine with the product (PLP-GABA*). This latter complex rearranges to enable transaldimination with the active site lysine (Lys276 in *Ec*GadB; E-PLP-GABA).^5^ The final step, leading to GABA release, regenerates the internal aldimine (E-PLP).

Previous studies of the wild-type *Ec*GadB measuring the ^13^C kinetic isotope effect in water at pL 4.7 reported a ^13^ *k*_cat_/*K*_M_ value of 1.018, significantly below the maximum theoretical value of 1.05–1.07. This suggests that the decarboxylation step is only partially rate-limiting. When this experiment was repeated in D_2_O, the [^13^ *k*_cat_/*K*_M_]^D_2_O^ value was 1.009, half that in water. This argues against a synchrony between the reaction of decarboxylation of l-Glu and protonation reaction of the quinonoid intermediate since the presence of deuterium in the solvent would not affect the ^13^C kinetic isotope effect in this situation. Rather, this represents a change in the partitioning factor to generate the catalytically competent orientation of α-carboxylate (PLP-Glu*). For the model above, *k*_cat_/*K*_M_ includes rate constants from the initial binding of the substrate to the first irreversible step, i.e., the decarboxylation step. In this study, the isotopic effect value on *k*_cat_/*K*_M_ (^D_2_O^ *k*_cat_/*K*_M_) for the reaction catalyzed by wild-type *Ec*GadB is approximately 1.7 (Table 2), and since we have ruled out a SKIE on the decarboxylation step, this isotope effect must reflect the solvent isotopically sensitivity of the steps leading to the catalytically competent species (PLP-Glu*), which must increase in D_2_O. This isotope effect value for ^D_2_O^ *k*_cat_/*K*_M_ is approximately the same as the solvent isotope effect observed for the ^13^C kinetic isotope effect, [^13^ *k*_cat_/*K*_M_]^D_2_O^. In the reaction catalyzed by the double variant GadB_D86N-H465A, the measured value of ^D_2_O^ *k*_cat_/*K*_M_ at pL 4.8 is retained (i.e., 2.12), strongly suggesting that Asp86 is not involved in substrate reorientation/conformational change at this pH.

In interpreting the ^D_2_O^ *k*_cat_, the model shows that *k*_cat_ includes rate constant contributions from steps from after the formation of the PLP-Glu* complex (i.e., under *k*_cat_ conditions, the substrate is saturating and no free enzyme is present) to the release of the final product. For the wild-type *Ec*GadB-catalyzed reaction, the value of ^D_2_O^ *k*_cat_ is 7–8, which is clearly too large to be accounted for by a single proton transfer step. The proton inventory is clearly not linear (Figure 5), suggesting that multiple proton transfers occur during the steps contributing to *k*_cat_, in agreement with previous findings.^13^ These events include proton transfers reported for ^D_2_O^ *k*_cat_/*K*_M_ (i.e., those occurring after l-Glu binding and prior to its decarboxylation), as well as additional proton transfers involved in product release, and likely arising from contributions of both. For the double variant GadB_D86N-H465A-catalyzed reaction, ^D_2_O^ *k*_cat_ is significantly reduced to 3 (Table 2), reflecting the solvent-isotopic sensitivity of the steps following decarboxylation and leading to product release. Since the substrate isomerization/conformational change for the double variant is less sensitive to solvent-isotopic composition, the product isomerization/conformational change would likely be equally insensitive, thus leaving only the reprotonation of the quinonoid intermediate and the E + PLP-GABA* transaldimination step, i.e., GABA release.

Notably, the decrease in the SKIE compared to that of the wild-type *Ec*GadB was observed only in the double variant and not in the single GadB_H465A variant, pointing to a prominent role of Asp86 in the solvent-isotopic sensitivity of *Ec*GadB. This was an unexpected finding because the Asp86-to-Asn mutation was expected to affect only substrate binding at neutral pH.

To conclude, the data presented in this work suggest that Asp86 has a surprising effect on the high SKIE observed in the l-Glu decarboxylation reaction catalyzed by wild-type *Ec*GadB. We propose that Asp86 plays a key role in both substrate binding and GABA release by altering the hydrogen-bonding network involved in substrate anchoring and product release. A nonoptimal initial interaction with the γ-carboxylate of l-Glu might explain the higher *K*_M_ value at acidic pH. However, following binding, l-Glu might be in a more favorable position for the subsequent reaction step (i.e., higher *k*_cat_). The kinetic constants and isotope effect discrepancies between the reactions catalyzed by GadB_D86N-H465A and both wild-type *Ec*GadB and GadB_H465A variants suggest that the above are likely possibilities. No significant differences in H_2_O and D_2_O were detected between wild-type *Ec*GadB and GadB_H465A, suggesting that His465 is involved in neither the binding of l-Glu nor in any of the catalytic steps, including decarboxylation and product release. This agrees with previously published suggestions.^6^

Interestingly, a recent study examined the role of Asp104 in the Gad from *Bacteroides fragilis* (*Bf*Gad), which corresponds to Asp86 in *Ec*GadB.^7,32^ In addition to slightly increasing the decarboxylation of l-Glu compared to the wild-type enzyme, the Asp104Asn substitution in *Bf*Gad significantly expanded the enzyme activity toward alternative substrates, such as l-aspartate, l-cysteinesulfinate, and l-homocysteate, whose decarboxylation yielded β-alanine, hypotaurine, and homotaurine, respectively. Although the wild-type enzyme was also found to be capable of decarboxylating these substrates, the removal of the negative charge of Asp104 in the *Bf*Gad D104N variant allowed the active site of this variant to better accommodate other substrates than l-Glu, underscoring the critical role of this residue, and of Asp86 in *Ec*GadB, by analogy, in substrate recognition and catalytic specificity.

Of interest is the finding that GadB_D86N-H465A can catalyze the l-Glu decarboxylation reaction at a significant rate at pH 7–8, where the activity of both GadB-H465A and wild-type *Ec*GadB is significantly lower and undetectable, respectively. It is important here to recall that l-Glu is a nonessential amino acid, abundant in many plant proteins, including those present in waste streams from biofuel production. This amino acid is therefore considered an interesting starting substrate for the industrial synthesis of nitrogen-containing chemicals, including GABA, a precursor to 2-pyrrolidone, an important industrial solvent.^33^ The enzymatic conversion of l-Glu to GABA via *Ec*GadB, carried out in bioreactors at the industrial level, was proposed as a strategy to decrease the dependence on fossil fuels, allowing the production of chemicals from renewable resources, such as biomass.^14^ However, during decarboxylation, *Ec*GadB activity is slowed down by the pH rise caused by the consumption of protons.^6,14^ Engineered variants of *E. coli* GadB, such as a C-terminal truncated mutant,^34^ His465 mutants,^6^ and Glu89/His465 mutants,^16^ were designed to improve and enhance the enzymatic properties of GadB and the reaction conditions for potential industrial applications. In this regard, GadB from lactic acid bacteria has also been the subject of extensive engineering efforts through directed evolution and (semi)­rational approaches. These strategies have aimed at broadening the pH range of GadB productivity and improving its thermal stability in order to enhance GABA yields under more favorable laboratory conditions. However, to our knowledge, Asp86 of *Ec*GadB, or the equivalent in Gads of lactic acid bacteria or other bacteria of biotechnological interest, has never been investigated in detail and considered for such purposes.^17,35–38^

## Conclusions

The GadB_D86N-H465A variant characterized in this work has allowed us to demonstrate that Asp86 is an unexpected contributor to the high SKIE in wild-type *Ec*GadB, likely through its impact on hydrogen-bond-mediated substrate binding and product release. In addition, the GadB_D86N-H465A variant is expected to be useful in biotechnological applications because, unlike *Ec*GadB and GadB_H465A, it is able to catalyze the decarboxylation reaction across a wide pH range, extending it to pH > 8.0, thereby rendering the reaction pH no longer a limiting parameter. It is important here to recall that *Ec*GadB has recently been shown to possess decarboxylase activity toward analogues of l-Glu, such as l-desmethylphosphinothrycin and l-homocysteinesulfinic acid, yielding the GABA analogues 3-aminopropylphosphinic acid and homohypotaurine, respectively.^39,40^ These findings clearly highlight the versatile nature of *Ec*GadB in decarboxylating substrates beyond l-Glu and pave the way for the use of the double variant characterized in this work to also improve the enzymatic synthesis of l-Glu analogues without pH being a limiting factor for their decarboxylation.

## †

Centro de Biología Molecular Severo Ochoa (CBM), CSIC—Universidad Autónoma de Madrid, Madrid 28049, Spain

## Abbreviations

GABA: γ-aminobutyrate
PLP: pyridoxal 5′-phosphate
SKIE: solvent kinetic isotope effect
